# Comparison of Robotic-Assisted vs. Conventional Laparoscopy for Para-aortic Lymphadenectomy in Gynecological Malignancies: A Systematic Review and Meta-Analysis

**DOI:** 10.3389/fsurg.2022.843517

**Published:** 2023-01-04

**Authors:** Zhengli Zhou, Jing Ge, Kefan Ye, Yifeng Zhang, Qian Hu, Limei Wang, Yiwen Chen

**Affiliations:** The First People's Hospital of Yunnan Province Gynecology Department (Affiliated Hospital of Kunming University of Science and Technology), Kunming, China

**Keywords:** robotic-assisted laparoscopy, extraperitoneal paraaortic lymphadenectomy, cervical cancer, conventional laparoscopy, gynecological malignancies

## Abstract

**Background:**

Robotic-assisted surgery is one of the novel minimally invasive surgical techniques for the treatment of gynecological malignancies. The aim of this systematic review and meta-analysis was to compare the outcomes of robot-assisted vs. conventional laparoscopy for para-aortic lymphadenectomy (PAL) in patients with gynecological malignancies.

**Methods:**

An electronic search in PubMed, Scopus, Cochrane Central Register of Controlled Trials (CENTRAL), and Google Scholar databases was performed for articles, published up to 01^st^ November 2021. Outcomes including operating time (OT), total blood loss (TBL), length of stay (LOS), and complication rate (CR) in robot-assisted vs. conventional laparoscopy were investigated.

**Results:**

A total of nine studies (7 non-RCTs and 2 RCTs) involving 914 participants were included. Of them, 332 patients underwent robotic laparoscopy (robotic group) and 582-conventional laparoscopy (conventional laparoscopy group). A significant decrease in TBL (MD = −149.1; 95% CI: −218.4 to −79.91) [ml] was observed in the robotic group as compared to the conventional laparoscopy group. However, no significant difference was noted for OT, CR, and LOS in the overall findings. Further subgroup analysis showed that the robotic group had a lower OT in mixed histological populations and studies reporting on the extraperitoneal approach. The lower chance of TBL was observed in mixed histological populations and studies involving extraperitoneal approach, Caucasian population, and non-RCTs design.

**Conclusions:**

Robotic laparoscopy has a significant advantage over the conventional laparoscopy approach for PAL in gynecological malignancies. Further prospective observational studies embedded with a large sample size are needed to validate our findings.

## Introduction

Para-aortic lymphadenectomy (PAL) is an important step in the surgical staging of a variety of gynecologic malignancies, such as cervical, endometrial, and ovarian cancers (1–3). It may provide significant information on the spread of cancer and the prognosis, and help in developing targeted primary and adjuvant therapy (4). However, complex surgical procedures like PAL might have substantial short- and long-term implications, such as the increased risk of intraperitoneal adhesions and radiation-related complications (1, 2). In 1997, Dargent et al. (5) proposed a method of using laparoscopic surgery for extraperitoneal lymphadenectomy. Laparoscopic extraperitoneal PAL has been proved to be safe and feasible in several trials (6–8) with the limitations of the typical transperitoneal laparoscopic method in terms of operative field exposure (due to obesity and overlaying bowel loops) (6, 7). Working in the retroperitoneal areas, on the other hand, can lead to complications and problems caused by ureteral, vascular, or intestinal disease (9). However, extraperitoneal laparoscopic PAL is still considered a difficult technique that necessitates a steep learning curve and advanced endoscopic abilities (6).

Common staging treatments for endometrial and ovarian malignancies include pelvic and para-aortic lymph node excision, hysterectomy, and bilateral salpingo-oophorectomy (10, 11). As a result, processes become lengthy and demanding. The emergence of robotic-assisted surgery has drastically changed gynecologic surgical practice in recent years (12–15). The use of robotic technology in laparoscopic surgery may overcome technical limitations of conventional laparoscopies, such as limited dexterity, flat two-dimensional vision, and difficulties in hand-eye coordination (16). Minimally invasive surgery for oncologic staging in endometrial and ovarian malignancies also results in a significant reduction in recovery time and length of hospital stay (17, 18). In the past few years, several studies have compared robot-assisted vs. conventional laparoscopy for PAL in patients with gynecological malignancies with limited data (8, 19–27). The main aim of this systematic review and meta-analysis was to compare the outcomes of robot-assisted vs. conventional laparoscopy for PAL in gynecological malignancies.

## Materials and Methods

### Study Protocol

This meta-analysis was done according to the Preferred Reporting Items for Systematic Reviews and Meta-Analyses (PRISMA) statement guidelines and the review protocol was registered on PROSPERO **CRD42021281371**.

### Eligibility Criteria

#### Inclusion Criteria

(a) Observational or interventional studies comparing the outcomes for patients with gynecological malignancies (endometrial cancer, cervical cancer, Ovarian Cancer) who underwent a para-aortic lymphadenectomy or both pelvic and para-aortic lymphadenectomy, by conventional laparoscopic and robotic approach; (b) Availability of at least one of the following outcome measures (operation time, total blood loss, length of hospital stay, and postoperative complications) in both robotic and conventional laparoscopy groups; (c) patients with gynecological malignancies, aged > 18 years.

#### Exclusion Criteria

(a) Duplicate studies, case series, case reports, systematic reviews, conference abstracts, preprints, and editorials; (b) Studies that do not describe relevant outcomes; (c) Full texts are unavailable.

### Outcome Measures

To assess operating time, total blood loss, length of stay in hospital, and complications rates in robot-assisted vs. conventional laparoscopy for PAL in gynecological cancers.

### Search Strategy

The following databases were searched for published articles up to November 1, 2021: PubMed, Scopus, Cochrane Central Register of Controlled Trials (CENTRAL), and Google Scholar. The following search terms were used: “robotics” OR “robot” OR “Conventional” AND “laparoscopy” AND “extraperitoneal” OR “retroperitoneal” AND “gynecological cancers” OR “gynecological malignancy” AND “lymphadenectomy” OR “para-aortic lymphadenectomy”. The titles and abstracts of the publications found in the initial search were examined. Studies were removed if they had no control group or only provided an abstract. Only Randomized Controlled trials (RCTs) and observational studies (prospective/retrospective cohort studies, case-controlled studies) were considered. Relevant studies were then identified and their full texts were independently examined in detail by the two reviewers. All disagreements were resolved in consensus with another reviewer. No language restriction was used during the literature search. References of the included papers were further searched for additional relevant studies.

### Data Extraction

A predefined data extraction table was used by two independents researchers to extract relevant information, including first author name, country, ethnicity, year of publication, study design, duration of the study, number of patients, treatment methods, population type, tumor grade, mean age, body mass index (BMI), operating time (OT), total blood loss (TBL), length of stay (LOS), postoperative complication rate (CR), and lymphadenectomy approach (transperitoneal/extraperitoneal). Ethnicity was categorized into Asian and Caucasian populations owing to a difference in the environmental, lifestyle, and cultural traditions in order to compare robotic-assisted vs. conventional laparoscopy for para-aortic lymphadenectomy in gynecological malignancies. Any disagreements were resolved by a discussion with the third researcher.

### Quality Assessment

The assessment of the quality of the included studies was conducted by using the Newcastle–Ottawa scale (NOS) (28). This assessment scale uses three broad factors (selection, comparability, and exposure), with the scores ranging from 0 (lowest quality) to 8 (best quality). Two authors independently rated the study's quality. Any disagreement was subsequently resolved by discussion or consultation with a third author.

#### Publication Bias

A funnel plot analysis was used to assess publication bias (29). Egger's regression test was used to determine the asymmetry of funnel plots (30).

#### Statistical Analysis

The analysis of continuous outcomes was done using the mean difference (MD) with a 95% CI. Dichotomous outcomes were analyzed using the risk ratios (RR) with 95 % CI. Values reported in median (range) were converted to mean [standard deviation (SD)] using an excel spreadsheet including all formulas that serve as comprehensive guidance for performing meta-analysis as described by Wan et al. (31). Cochran's Q- and *I*^2^-tests were used to assess heterogeneity among outcomes of the included studies. When *I*^2^ was > 50%, some degree of heterogeneity was assumed, and the random-effects model was employed. In all other cases, the fixed effects model was utilized. Subgroup analysis based on ethnicity, types of study, histological type, and lymphadenectomy approach (transperitoneal/extraperitoneal/both) was done for all the included studies. Selection bias and heterogeneity arising from individual studies were assessed by sensitivity analyses. This involved the sequential deletion of a single study during each turn. A *P*-value of < 0.05 was considered statistically significant. All statistical analyses were performed using Review Manager version 5.3 software (Nordic Cochrane Center, Copenhagen, Denmark).

## Results

### Literature Selection

**Figure 1:**
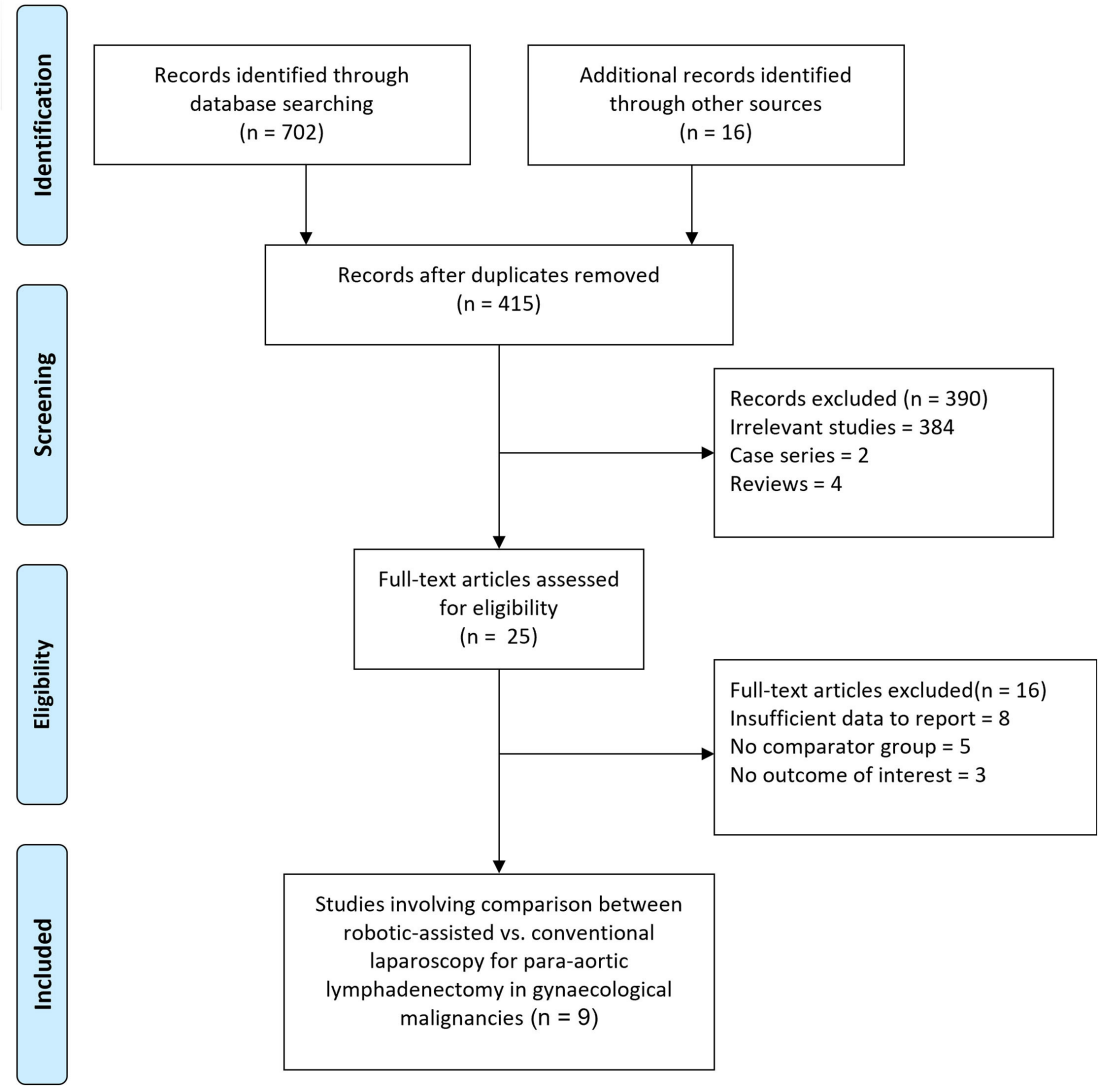
Flow diagram for the selection of studies and specific reasons for exclusion from the present meta-analysis.

The initial search generated 718 records, of which 415 records remained after duplicates were removed. As summarized in Figure 1, following the exclusion of irrelevant studies and review articles, 25 eligible articles were further evaluated for eligibility. Finally, after evaluating study details, 16 articles were removed due to insufficient data or overlapping data, leaving the current meta-analysis with 09 studies (8, 19–26). PRISMA 2020 checklist has been provided in the Supplementary Table S1.

### Study Characteristics

A total of nine studies [7 non-RCTs (8, 19, 21–25) and 2 RCTs (20, 26)] involving 914 participants (332 patients in the robotic group and 582 in the conventional laparoscopy group) were included in our systematic review and meta-analysis. All included studies were published between 2014 and 2021 and the sample size in the studies ranged from 17 to 162 patients with gynecological malignancies. Of nine studies, six (8, 19, 21–24) were of retrospective cohort and only one study was prospective (25). Six studies were conducted in the Caucasian population (8, 20, 21, 24–26) while three studies were in patients of Asian ethnicity (19, 22, 23). Five studies included patients with endometrial cancer (8, 19, 20, 22, 23), two studies included patients with cervical cancer (21, 24), and two studies included patients with endometrial, ovarian, and cervical cancer (25, 26). The baseline, clinical characteristics, and quality score of the included studies are summarized in Table 1. Most studies were of good quality, with a NOS score of six or higher. PRISMA checklist has been provided in the Supplementary Table S1.

**Table 1:** Characteristics of included studies for the comparison of robotic-assisted vs. conventional laparoscopy for para-aortic lymphadenectomy in gynecological malignancies.

**S. No**.	**References**	**Country**	**Study** **period**	**Study** **design**	**Histological** **type**	**Groups**	**Sample** **size**	**Approach**	**Tumor** **grade** **(*n*) I/II/III**	**Age**	**Inclusion** **criteria**	**Exclusion** **criteria**	**NOS score**
1.	Torng et al. (19)	Taiwan	Jan 2012 -Oct 2015	RC	Endometrial Cancer	LSS RSS	24 20	Transperitoneal	20/3/1 14/3/3	57 ± 5.5 56.4 ± 7.7	Cases of LSS or RSS for the indication of endometrial cancer by a local board certified gynecology oncologic surgeon	NA	6
2.	Salehi et al. (20)	Sweden	May 2013 -July 2016	RCT	Endometrial Cancer	LT RALS	48 48	Transperitoneal	43/5/0 42/6/0	61.5 ± 8 65.2 ± 5.1	(1) aged between 18 and 75 years, (2) histologically confirmed endometrial cancer, presumed International Federation of Gynecology and Obstetrics (FIGO) stage I or II.	(1) Ongoing anti-tumor treatment (except treatment with tamoxifen or aromatase inhibitors) (2) pre-operative imaging indicating extrauterine spread, medically unfit for extensive surgery (3) disseminated disease diagnosed during surgery or inability to comply to the protocol.	6
3.	Loverix et al. (21)	Belgium	Dec 1994 -Dec 2016	RC	Cervical cancer	LPAO	162	Trans- or extra-peritoneal	29/107/26	49 ± 10.5	Locally advanced cervical cancer patients (FIGO 2009 stage IB2-IVA or IB1 with suspicious pelvic lymph nodes) who underwent a para-aortic lymphadenec tomy up to the inferior mesenteric artery, by either laparoscopic or robotic approach	Presence of other primary malignancies, para-aortic lymphadenectomy combined with other surgery (such as hysterectomy, pelvic lymphadenectomy or omentectomy), prior radiotherapy or retroperitoneal surgery, metastatic disease outside of the pelvis on preoperative imaging, poor general condition of the patient, and inoperability due to intraperitoneal adhesions.	6
						RPAO	55		9/39/7	49.7 ± 12.5			
4.	Lee et al. (22)	Korea	June 2006- Oct 2016	RC	Endometrial Cancer	R L	26 16	Transperitoneal	7/15/4 4/11/1	56.7 ± 6.9 51.1 ± 7.8	Surgical management included total hysterectomy with removal of both adnexa and bilateral pelvic, infrarenal para-aortic lymph node dissection. Aortic node dissection was extended up to the level of the renal vein. Endometrial cancer was staged according to the current guidelines approved by the International Federation of Gynecology and Obstetrics.	NA	7
5.	Lee et al. (23)	Korea	June 2006- Oct 2016	RC	Endometrial Cancer	R L	47 43	Transperitoneal	34/11/2 27/9/7	53.5 ± 8.6 48.4 ± 10.4	Indications for TIPAL were staging of ovarian cancer in the early stage, staging of high-risk endometrial cancer, and evaluation of the status of the paraaortic nodes to adjust the radiation fields in locally advanced cervical cancer. Disease was staged in accordance with the current guidelines approved by the International Federation of Gynecology and Obstetrics	NA	7
6.	Feijoo et al. (24)	Spain	July 2009- Jan 2013	RC	Cervical Cancer	R L	17 83	Extraperitoneal + Transperitoneal	5/9/3 30/42/11	48.2 ± 10. 50 ± 10.2	Non-consecutive patients with locally advanced cervical cancer (FIGO stages IB2, IIA2 and IIB– IVA), All patients underwent robotic-assisted laparoscopic extraperitoneal paraaortic and common iliac lymphadenectomy	Severe cardiorespiratory disease, age 80 years old or older, prior radiotherapy or retroperitoneal surgery and evidence of metastatic disease outside of the pelvis in preoperative imaging study.	6
7.	Coronado et al. (25)	USA	Jan 2010- June 2013	PC	Ovary Cervix Endometrium	R L	32 30	Transperitoneal	22/10 26/4	57.3 ± 4.6 55.7 ± 6.7	(1) Disease was staged according to the current guidelines approved by the Spanish Society of Gynecology and Obstetrics. (2) All patients with gynecologic malignancies that required TIPAL for staging.	Patients who had undergone primary surgery with para-aortic lymph node recurrence and those who had received previous chemotherapy and/or radiotherapy	6
8.	Bebia et al. (26)	Spain	June 2012- Jan 2019	RCT	Ovary Endometrium	R	35	Extraperitoneal	3/17/15	64.1 ± 7.	Patients diagnosed with either initial-stage endometrial cancer (patients with tumors invading ≥50% of the myometrium, elicited by MRI and/or transvaginal ultrasonography; cervical stromal involvement; grade 3 endometrial tumors; or non-endometrioid tumors) or ovarian malignancy with an indication of surgical staging	Previous PALND, pelvic and/or aortic radiotherapy, or perioperative suspicion of advanced stage disease.	8
						L	68		5/25/38	63.6 ± 11.3			
9.	Pakish et al. (8)	USA	Jan 2007- Nov 2012	RC	Endometrial Cancer	R L	52 108	Extraperitoneal	40/6/6 71/14/23	60 ± 9. 59.5 ± 13.4	Patients who underwent extraperitoneal laparoscopic or transperitoneal laparoscopic or robotic para-aortic lymphadenectomy for staging of endometrial cancer	NA	8

PC, Prospective Cohort; R, Retrospective Cohort; RCT, Randomized Control trial; LN, Lymph Node; LSS, laparoscopic; RSS, Robotic-assisted staging surgery; RALS, Robot-assisted laparoscopic surgery; LT, laparotomy; BMI, body mass index; FIGO, International Federation of Gynecology and Obstetrics; LPAO, laparoscopic para-aortic lymphadenectomy; RPAO, robotic para-aortic lymphadenectomy; R, Robot; L, Laparoscopy; T, Traditional; NOS, Newcastle Ottawa Scale.

### Operation Time

**Figure 2:**
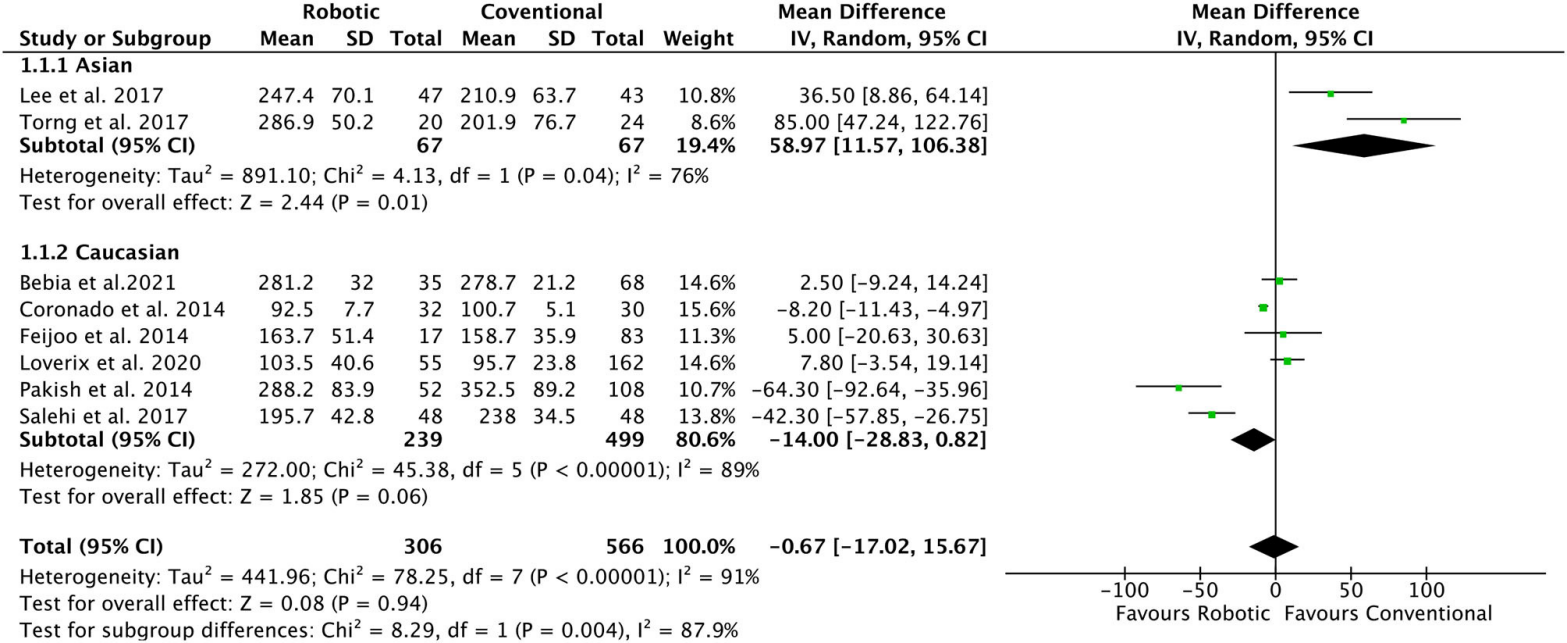
Forest plot of the association of operation time in robotic-assisted vs. conventional laparoscopy for para-aortic lymphadenectomy in gynecological malignancies.

Eight studies (8, 19–21, 23–26) with a total of 872 patients reported the data for OT (mins) in both robotic and conventional laparoscopy groups. No significant changes were observed in operative time for overall population (MD = −0.67, 95% CI: −17.02 to 15.67) (Figure 2). The random-effect model was applied in the analyses as significant heterogeneity (*I*^2^ = 91%, *p* = <0.0001) was observed. Stratified analysis based on ethnicity, types of study, histological type and lymphadenectomy approach, showed significantly shorter operation time in studies with extraperitoneal approach (MD = −24.4, 95% CI: −44.9 to −3.97, *I*^2^ = 92%) (Table 2). However, more operation time was required in the robotic group as compared with the conventional laparoscopy group for studies conducted in the Asian population and with a transperitoneal approach (MD = 58.97, 95% CI: 11.57 to 106.38, *I*^2^ = 76%).

**Table 2:** Summary of effect size based on subgroup analysis for the comparison of robotic-assisted vs. conventional laparoscopy for para-aortic lymphadenectomy in gynecological malignancies.

**Variables**	**Subgroups items**	**No. of studies**	**Operation time MD [95% CI]**	**Total blood loss MD [95% CI]**	**Length of stay MD [95% CI]**	**Complication rate RR [95% CI]**	**References**
Histological type	Endometrial cancer	05	2.40 [−56.63, 61.43]	−101.20 [−334.07, 131.67]	1.60 [−0.07, 3.27]	0.93 [0.40, 2.16]	(8, 19, 20, 22, 23)
	Cervical cancer	02	7.34 [−3.03, 17.71]	−501.66 [−1460.77, 457.44]	−0.04 [−0.82, 0.74]	0.98 [0.19, 5.01]	(21, 24)
	Mixed (Ovary, Cervix and Endometrium)	02	−4.40 [−14.44, 5.64]	−27.05 [−43.01, −11.08]	0.21 [−1.36, 1.77]	0.48 [0.11, 1.97]	(25, 26)
Ethnicity	Asian	03	58.97 [11.57, 106.38]	−28.27 [−69.75, 13.20]	1.32 [−1.40, 4.04]	1.22 [0.51, 2.88]	(19, 22, 23)
	Caucasian	06	−14.00 [−28.83, 0.82]	−216.87 [−303.29, −130.46]	0.49 [−0.44, 1.41]	0.69 [0.33, 1.47]	(8, 20, 21, 24–26)
Approach	Transperitoneal	02	58.97 [11.57, 106.38]	−28.20 [−69.85, 13.45]	1.32 [−1.40, 4.04]	1.22 [0.51, 2.88]	(19, 20, 22, 23, 25)
	Extraperitoneal	05	−24.47 [−44.98, −3.97]	−80.89 [−154.27, −7.50]	0.85 [−0.45, 2.15]	0.58 [0.24, 1.39]	(8, 26)
	Extraperitoneal + Transperitoneal	02	7.34 [−3.03, 17.71]	−501.66 [−1460.77, 457.44]	−0.04 [−0.82, 0.74]	0.98 [0.19, 5.01]	(21, 24)
Study design	Non-RCTs	07	−19.60 [−63.50, 24.30]	83.43 [−153.90, 320.76]	1.57 [0.39, 2.74]	0.85 [0.05, 13.86]	
	RCTs	02	7.23 [−14.21, 28.67]	−245.82 [−403.40, −88.23]	0.03 [−0.63, 0.69]	0.81 [0.45, 1.43]	(20, 26)

RCTs, Randomized Control trial; MD, Mean Difference; CI, Confidence Interval.

*Bold values represent statistically significant results (p < 0.05).

### Total Blood Loss

**Figure 3:**
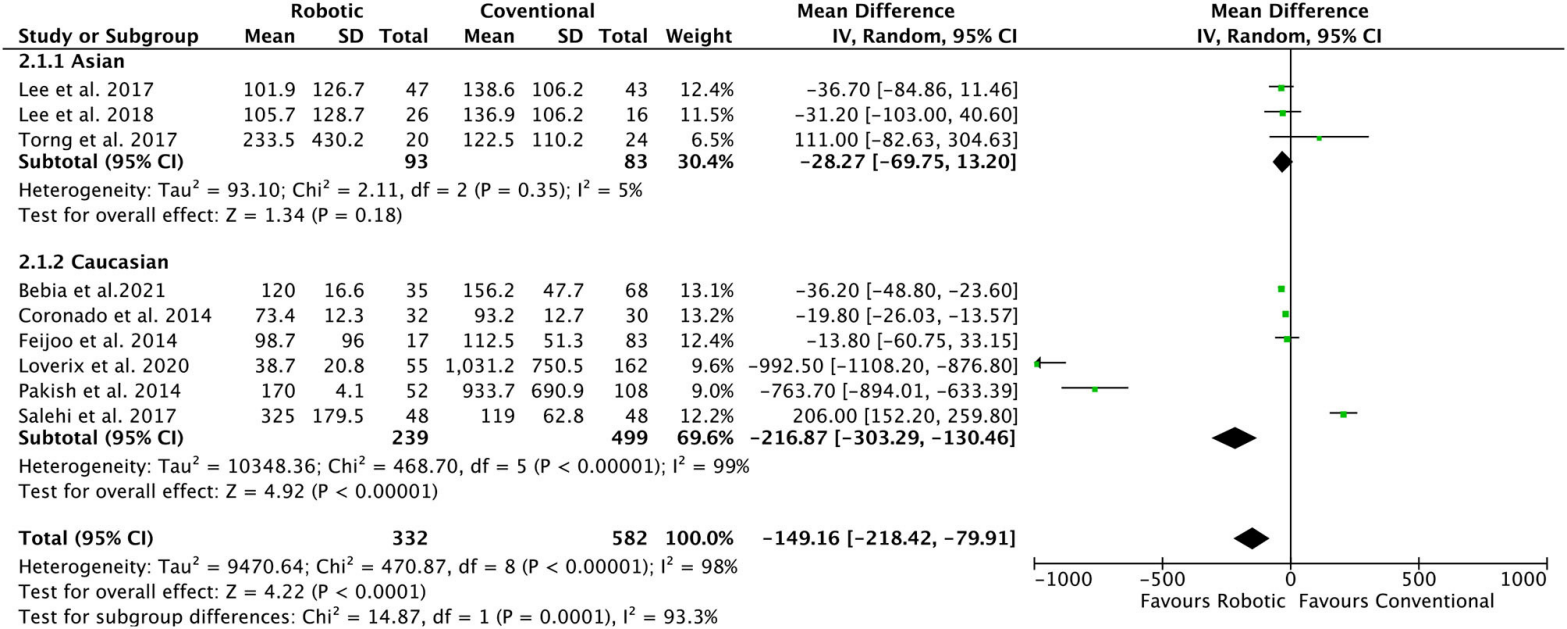
Forest plot of the association of total blood loss (ml) in robotic-assisted vs. conventional laparoscopy for para-aortic lymphadenectomy in gynecological malignancies.

All included studies (8, 19–26) reported the data for the perioperative TBL (ml). Data showed an overall lower chance of TBL in robotic as compared to conventional laparoscopy group (MD = −104.2, 95% CI: −218.49 to −79.91; *I*^2^ = 98%) (Figure 3). Subgroup analyses suggested a lower chance of TBL in mixed histological population and studies involving extraperitoneal approach (MD = −216.8, 95% CI: −303.3 to −130.4; *I*^2^ = 99%), Caucasian population (MD = −80.9, 95% CI: −154.2 to −7.5; *I*^2^ = 98%), and non-RCT study design (MD = −245.8, 95% CI: −403.4 to −88.2; *I*^2^ = 98%). However, no significant changes in TBL were detected in the robotic group as compared to the conventional laparoscopy group in Asian studies, RCTs studies, transperitoneal approach, and histological types studies (Table 2).

### Length of Stay

A total of seven studies (19–22, 24–26) with a total of 664 patients reported the data for the LOS in hospital (days). No significant changes in the LOS were observed in robotics as compared with the conventional laparoscopy group (MD = 0.62, 95%CI: −0.19 to 1.4; *I*^2^ = 95%) (Table 2). Subgroup analysis based on ethnicity, types of study, histological type, and lymphadenectomy approach also showed a non-significant change in LOS.

### Complication Rate

A total of nine studies (8, 19–26) with 914 patients reported a non-significant change in postoperative complication rate (RR = 0.80, 95% CI:0.45 to 1.42; *I*^2^ =45%) in the robotic group as compared to the conventional laparoscopy group (Table 2). Based on ethnicity, stratified analysis also showed a non-significant change in postoperative complication rate (RR = 0.69, 95% CI:0.33 to 1.47; *I*^2^ = 56%) in both Caucasian as well as in Asian population (RR = 1.22, 95% CI:0.51 to 2.88; *I*^2^ = 0%).

### Sensitivity Analysis and Publication Bias

**Figure 4:**
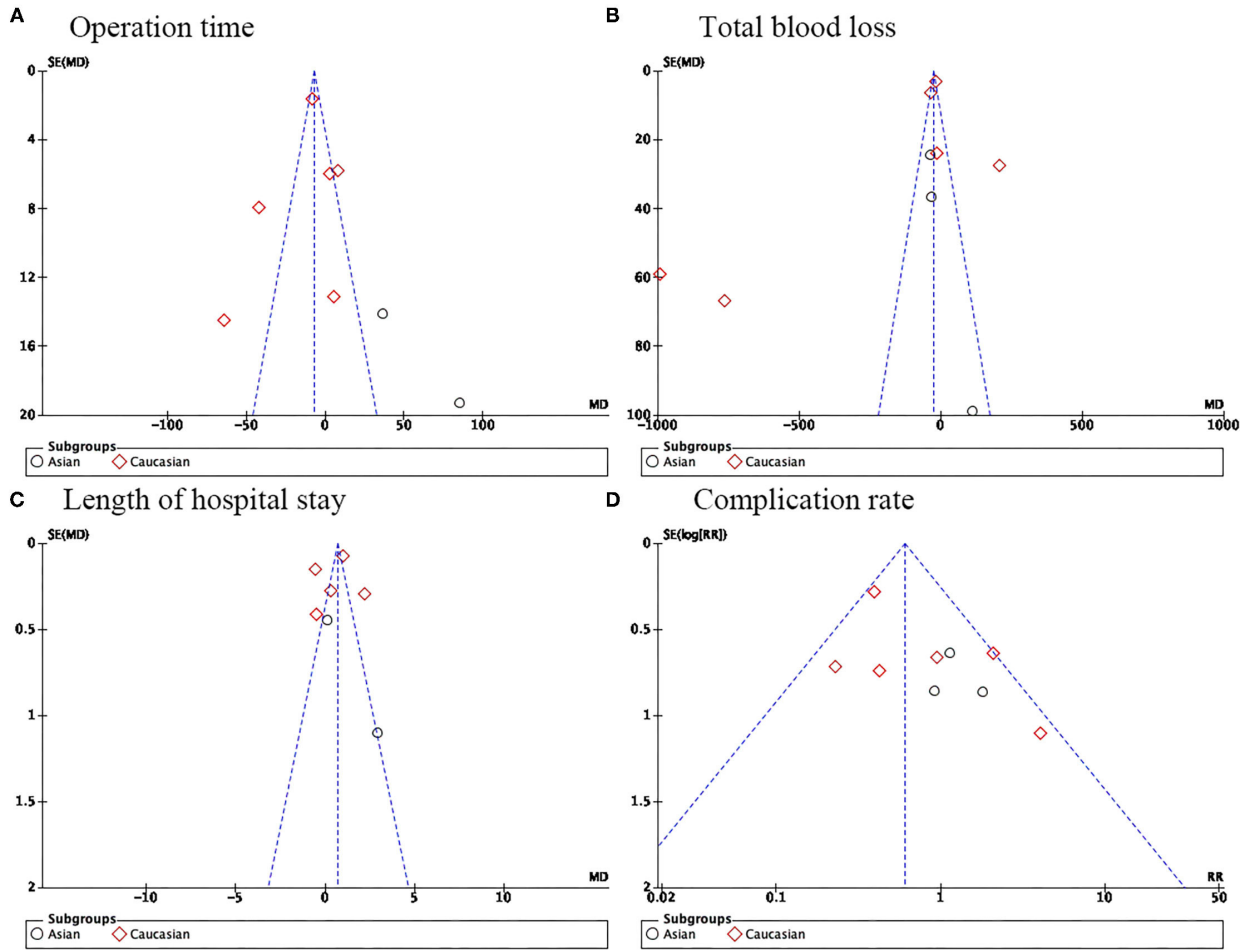
Begg's funnel plot of publication bias testing for the association of **(A)** operation time, **(B)** total blood loss, **(C)** length of hospital stay, and **(D)** complication rate in robotic-assisted vs. conventional laparoscopy for para-aortic lymphadenectomy in gynecological malignancies.

A sensitivity analysis was performed using the leave-one-out method. This method re-evaluates the pooled effect size of the included studies by removing a single study at a time. A significant change in the effect size, measuring the association change in postoperative in the robotic group as compared to the conventional laparoscopy group, was observed when an outlier study by Salehi et al. was removed (20). No significant evidence of publication bias was found. An asymmetrical inverted funnel plot for OT, TBL, LOS, and CR was observed (Figure 4).

## Discussion

The strengths of our study include the fact that, to our knowledge, this is the first systematic review and meta-analysis that compared the robotic vs. conventional laparoscopy approaches for PAL in gynecological maliginacies. In our meta-analysis, a significant decrease in TBL was observed in robot-assisted as compared to conventional laparoscopy. However, no significant differences were noted for OT, CR, and LOS in the overall findings. More operation time was required in the robotic group as compared with the conventional laparoscopy group for studies conducted in the Asian population vs. Caucasian Population and with a transperitoneal approach as compared to the extraperitoneal approach. Further subgroup analysis demonstrated that the robotic group had a lower OT in the mixed histological population and in studies with an extraperitoneal approach. The lower chances of TBL were observed in mixed histological populations and studies involving extraperitoneal approach, Caucasian population, and non-RCT study design. Our findings may aid surgeons in the decision-making for the best surgical approach to treat of gynecological malignancies.

Previous research on the role of extraperitoneal laparoscopic PAL in gynecologic malignancies has demonstrated that this operation is both safe and practical (7, 32, 33). This method has been proven to have few problems and a low likelihood of converting to transperitoneal laparoscopic or open lymphadenectomy (7, 32). A study by Bebia et al. (26) compared transperitoneal or extraperitoneal laparoscopic and transperitoneal robot-assisted approaches to performing PAL. Their study showed that the extraperitoneal robotic approach resulted in fewer complications, even in older patients with high BMI. All examined approaches did not show any differences in aortic lymph node count, operative time, or LOS. Elderly patients affected by gynecological cancer should benefit from individualized treatment. Data, in fact, do not support the premise that age itself is a negative prognostic factor, moreover with the objectivity that elderly patients are able to tolerate the standard of care for gynecological cancers. In this perspective, it is absolutely necessary to overcome the mental bias of not treating the elderly because they are more fragile and have a lower life expectancy than their younger counterparts (34). The advanced technology of robotic-assisted laparoscopy gives advantages for performing these difficult surgical procedures. Many studies had reported their initial experience with robotic surgery and showed its feasibility in endometrial cancer. Postoperative complications were comparable and in contrast to previous retrospective studies, the total health care cost was significantly lower for robotic surgery. The choice of surgical treatment modality will ultimately depend on patient/surgeon preference and institutional resources. Further development of robotic surgery such as intra-operative high-quality navigation and imaging systems, could open fascinating new avenues for PAL in gynecological malignancies.

Another study by Pakish et al. (8) compared the outcomes of extraperitoneal laparoscopic PAL and transperitoneal minimally invasive (laparoscopic or robotic) PAL. They also concluded that extraperitoneal PAL is associated with fewer complications and lower failure rates than transperitoneal PAL, although it is associated with longer operational periods. Our subgroup analyses showed that extraperitoneal robot-assisted approaches had a lower operative time and lesser chance of blood loss (8, 26). We couldn't perform additional analyses for the survival outcome, conversion to laparotomy, change in post-operative hemoglobin concentration and post-operative hospitalization due to the unavailability of data in the included studies (21–23). Additionally, data for the complications using different grades based on the Clavein dindo classification was available for only two studies by Loverix et al. and Salehi et al. for which analysis was not possible (20, 21).

The infrarenal para-aortic area is difficult to approach due to several limitations of the conventional laparoscopic approach, including a steep learning curve due to the unique surgical skills required, high reliance on skilled surgical assistants, the condition of the patient (morbid obesity), or significant intra-abdominal adhesions (35–37). The robotic-assisted approach took less time per infrarenal para-aortic and total lymph nodes retrieved compared to the conventional laparoscopic approach (22). The primary benefits of robotic technology in minimally invasive surgery include enhanced accuracy and precision, articulation of robotic instruments, stereoscopic picture, and surgeon's sitting position (9). Robotic-assisted and conventional laparoscopy provide similar perioperative outcomes other than lower blood loss and a higher number of aortic nodes removed (both without clinical impact) in robotic patients for the performance of extraperitoneal paraaortic lymphadenectomy in patients with locally advanced cervical cancer (24). Pelvic lymphadenectomy involves an extensive compromise of the whole pelvic lymphatic system compared to the removal of the Sentinel lymph node alone. The pelvic nodes are easily accessible using a transperitoneal technique, while the paraaortic nodes are more difficult to reach. Access to the paraaortic nodes is mostly determined by the patient's weight and the surgeon's experience. Since the intestines and omentum retain so much fat, para-aortic lymphadenectomy is more challenging in obese patients. To access the para-aortic nodes, these structures must be mobilized and retracted out of the dissection field. Even in obese patients, an extraperitoneal technique provides excellent exposure to the para-aortic nodes. However, unless additional port sites are implanted, a pelvic lymph node dissection below the level of the common iliac nodes is not conceivable (10, 38). Robotic single-site pelvic lymphadenectomy using bipolar forceps and the monopolar hook is feasible. New developments are needed to improve surgical ergonomics and additional studies should be performed to explore possible benefits of this procedure (39–41).

Our meta-analysis must be interpreted with caution as there are limitations to our findings: (1) The included papers lacked long-term follow-up results, such as the rate of local tumor recurrence or distant metastasis, and survival rate. Only two studies were RCTs, increasing the probability of substantial implementation bias. None of the studies indicated whether outcome evaluators were blinded. That may result in possible measurement bias, especially in subjective outcomes such as length of stay. (2) Sample sizes in the included studies were small. Six out of nine included studies were retrospective which significantly increases the risk of bias for under-reporting complications, especially minor complications. (3) Study participants were at diagnosed with various phases and types of gynecological malignancies and their progression, a parameter which cannot be controlled. (4) Lastly, a high degree of heterogeneity was observed in the overall analysis, possibly due to different study designs, differences in patient populations and surgeons, discharge criteria, and hospital policies regarding the post-operative stay.

## Conclusions

Robotic laparoscopy has a significant advantage over the conventional laparoscopy approach for PAL in gynecological malignancies. Further prospective observational studies embedded with a large sample size are needed to validate our findings.

## Data Availability Statement

Publicly available datasets were analyzed in this study. This data can be found at: PubMed Central, Cochrane library, EMBASE, and MEDLINE databases from inception until May 2021 for relevant publications.

## Author Contributions

ZZ and JG conceived and designed the study. KY, YZ, QH, and LW were involved in literature search and data collection. ZZ, JG, and YC analyzed the data. YC wrote the article and reviewed and edited the manuscript. All authors read and approved the final manuscript.

## Funding

This study was supported by Yunnan Provincial Department of Science and Technology—Project number: 2019FE001 (-296).

## Conflict of Interest

The authors declare that the research was conducted in the absence of any commercial or financial relationships that could be construed as a potential conflict of interest.

## Publisher's Note

All claims expressed in this article are solely those of the authors and do not necessarily represent those of their affiliated organizations, or those of the publisher, the editors and the reviewers. Any product that may be evaluated in this article, or claim that may be made by its manufacturer, is not guaranteed or endorsed by the publisher.
